# Disease activity and patient-reported outcomes in patients with rheumatoid arthritis and Sjögren’s syndrome enrolled in a large observational US registry

**DOI:** 10.1007/s00296-020-04602-8

**Published:** 2020-05-24

**Authors:** Leslie R. Harrold, Ying Shan, Sabrina Rebello, Neil Kramer, Sean E. Connolly, Evo Alemao, Sheila Kelly, Joel M. Kremer, Elliot D. Rosenstein

**Affiliations:** 1 Corrona, LLC, 1440 Main Street, Suite 310, Waltham, MA 02451 USA; 2 University of Massachusetts Medical School, Worcester, MA USA; 3 Institute for Rheumatic & Autoimmune Diseases, Overlook Medical Center, Summit, NJ USA; 4 Bristol Myers Squibb, Princeton, NJ USA; 5 Albany Medical College and the Center for Rheumatology, Albany, NY USA

**Keywords:** Sjögren’s syndrome, Rheumatoid arthritis, Treatment response, Disease activity, Patient-reported outcomes, ACPA

## Abstract

The objective of this study was to compare rheumatoid arthritis (RA) disease activity and patient-reported outcomes (PROs) in a national sample of patients with RA with/without Sjögren’s syndrome (SS). Adults with RA from a large observational US registry (Corrona RA) with known SS status between 22 April 2010 and 31 July 2018 and a visit 12 (± 3) months after index date were identified (*n* = 36,256/52,757). SS status: determined from a yes/no variable reported at enrolment into the Corrona RA registry and follow-up visits. Index date: date that SS status was recorded (yes/no). Patients received biologic or targeted synthetic disease-modifying antirheumatic drugs as part of standard care. Patients with RA only were followed for ≥ 12 months to confirm the absence of SS. Patients were frequency- and propensity-score matched (PSM) 1:1 and stratified by disease duration and treatment response-associated variables, respectively. Clinical Disease Activity Index (CDAI) and PROs 12 months after index visit were compared in patients with and without SS. Baseline characteristics in 283 pairs of PSM patients were balanced. Mean change in CDAI score was numerically lower in patients with RA and SS than patients with RA only (8.8 vs 9.3). Reductions in PROs of pain, fatigue and stiffness were two- to threefold lower for patients with RA and SS versus RA only. Reductions in RA disease activity and RA-related PROs were lower in patients with RA and SS versus those with RA only. Our data indicate that SS adds to treatment challenges; physicians may wish to consider SS status when managing patients with RA.

**Electronic supplementary material:**

The online version of this article (10.1007/s00296-020-04602-8) contains supplementary material, which is available to authorized users.

## Introduction

Sjögren’s syndrome (SS) is a systemic autoimmune disease that can occur independently or in conjunction with another autoimmune condition, such as rheumatoid arthritis (RA) [1, 2]. SS has traditionally been thought of as either primary (SS only) or secondary; however, the terminology for secondary SS has recently evolved to be more descriptive, particularly because SS and other autoimmune diseases are co-existing conditions, rather than one being secondary to the other [2].

The current American College of Rheumatology (ACR) and European League Against Rheumatism (EULAR) treatment guidelines for RA and the ACR/EULAR classification criteria for SS do not include recommendations for treating patients with both RA and SS [3–5]. However, patients with both RA and SS have an increased disease burden [6, 7] and a decreased quality of life [8] compared with patients with only one autoimmune disease. For example, patients with RA and SS are more likely to have a longer duration of RA and worse joint damage compared with patients with RA only [6, 9]. Patients with SS alone are more likely to experience a negative impact on physical functioning (e.g., lifting and carrying, climbing stairs, bending and walking moderate distances) and social functioning (e.g., quality and quantity of social activities with others) than control subjects without dry eye disease or SS [8, 10].

The correlation of SS with increased disease burden in patients with RA includes an association with higher RA disease activity and anti-citrullinated protein antibody (ACPA) positivity (+) [6, 11]. ACPAs play a pivotal role in the progression of RA [11, 12], indicating a poor prognosis [4], more severe disease course and radiological destruction compared with patients with RA who are ACPA negative (−) [13]. However, there are limited data on the impact of ACPA positivity and SS status in patients with RA and SS [6, 9, 14, 15].

Evaluating real-world data from patients with RA and SS and patients with RA only may enable clinicians to understand the different needs of these populations. The primary objective of this study was to compare RA disease activity and patient-reported outcomes (PROs) in patients from a national registry sample of patients with RA, with and without SS. A secondary objective was to compare RA disease activity and PROs in a sub-group of ACPA+ patients with RA, as measured by anti-cyclic citrullinated peptide (anti-CCP), with and without SS.

## Materials and methods

### Data source

The Consortium of Rheumatology Researchers of North America (Corrona) RA registry is an independent, prospective, national, observational cohort in which standardized and uniform treatment and outcome data are collected from treating rheumatologists at the time of a clinical encounter for treatment of patients with RA. Patients have been recruited from 182 private practices and academic sites across 42 US states, with 781 participating rheumatologists. As of June 2019, the Corrona RA registry included information on 52,757 patients. Data on 397,236 patient visits and approximately 188,161 patient-years of follow-up observation time have been collected, with a mean patient follow-up of 4.5 (median 3.3) years. The characteristics of the Corrona registry have been described previously [16].

This study was carried out in accordance with the Declaration of Helsinki. All participating investigators were required to obtain full institutional review board (IRB) approval for conducting non-interventional research involving human subjects. Sponsor approval and continuing review was obtained through a central IRB (New England Independent Review Board, NEIRB No. 120160610). For academic investigative sites that did not receive a waiver to use the central IRB, full board approval was obtained from the respective governing IRBs and documentation of approval was submitted to Corrona, LLC, prior to initiating any study procedures. All registry patients were required to provide written informed consent and authorization prior to participating.

### Study population

**Fig. 1:**
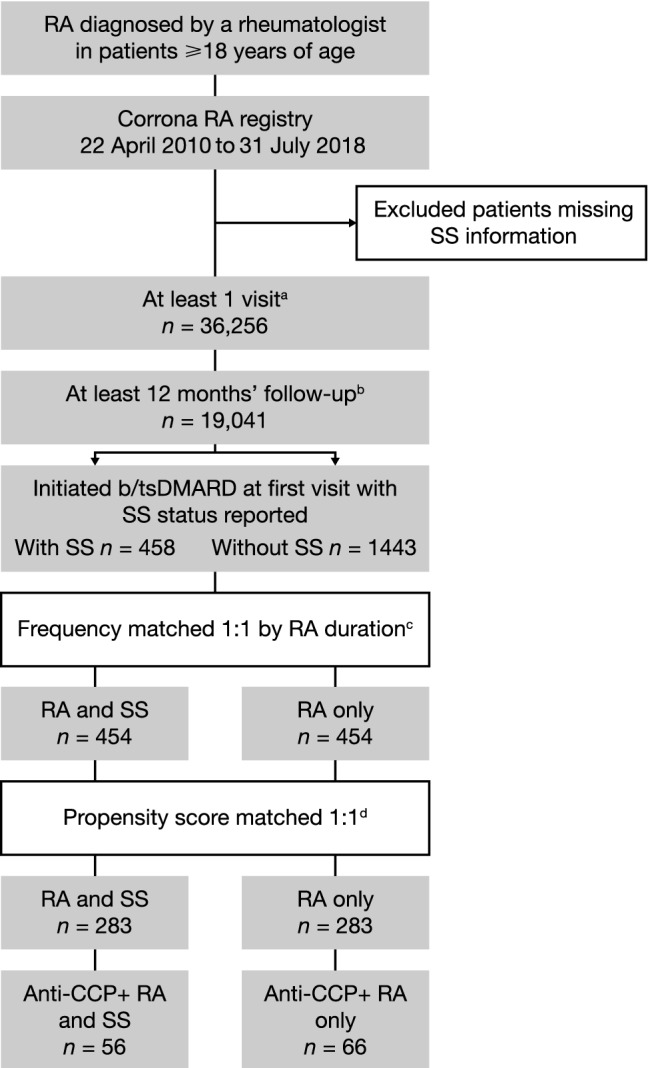
Selection of eligible patients for analysis. ^a^Yes/no to having SS. ^b^After the first capture of SS data in patients with a diagnosis of no SS. ^c^Between patients with and without SS by biologic initiators. ^d^Informed from frequency matching, based on logistic regression model predicting group (RA and SS versus RA only). *b/tsDMARD* biologic or targeted synthetic disease-modifying antirheumatic drug, *CCP+* cyclic citrullinated peptide positive, *RA* rheumatoid arthritis, *SS* Sjögren’s syndrome

This study included adult patients with rheumatologist-diagnosed RA enrolled in the Corrona RA registry between 22 April 2010 and 31 July 2018 (Fig. 1). SS status was captured by physicians at enrolment and follow-up visits using a provider form that included a yes/no question regarding the status of SS associated with RA. The presence of SS was determined clinically based upon the presence, or absence, of symptomatic dry eyes and/or mouth judged by the treating physician not to be related to medications. The index date was defined as the date the provider first reported SS status as yes (patients with RA and SS) or no (patients with RA only). Data were included if patients had at least one visit assessing SS status (SS associated with RA: yes/no) and at least 12 months of follow-up after index date. If data were available for more than one visit, the visit closest to the 12-month post-index date was used. Both patients with and without SS were required to have initiated a biologic (b) or targeted synthetic (ts) disease-modifying antirheumatic drug (DMARD). Patients without b/tsDMARD initiation or with missing SS information were excluded.

### Study assessments

Patients were randomly frequency matched 1:1 (patients with RA and SS and patients with RA only) based on the duration of RA (< 2, ≥ 2 to < 5, ≥ 5 to < 10, ≥ 10 to < 15, ≥ 15 to < 20, ≥ 20 to < 30 and ≥ 30 years). Due to residual imbalance after frequency matching, frequency-matched patients were pooled together and propensity-score matched (PSM) 1:1 based on a logistic regression model-predicting group (RA and SS versus RA only). In the propensity score model, baseline demographics and response characteristics were compared between the cohorts (RA and SS versus RA only), and the absolute values of the standardized differences were estimated. The absolute value of the standardized difference of ≤ 0.1 for the overall population [17] was taken to indicate a negligible difference in the mean or prevalence of a covariate between cohorts; variables with a standardized difference > 0.1 were included in the construction of the propensity score model.

Variables assessed included demographic and socioeconomic characteristics, lifestyle factors, history of comorbidities, RA disease characteristics, previous and current RA therapy, RA disease activity and PROs. Patients with an SS diagnosis (patients with RA and SS) were compared with patients without an SS diagnosis (patients with RA only). For the subgroup analysis in patients who were anti-CCP+, anti-CCP testing was performed by investigators and anti-CCP+ was defined as ≥ 20 units/mL. Statistical significance was estimated using Student’s *t* test for continuous variables and Chi-square test for category variables.

### Study outcomes

The primary outcome was the mean change in Clinical Disease Activity Index (CDAI) score from index visit to Month 12 in PSM patients [18]. The secondary outcome was mean change in PROs (modified Health Assessment Questionnaire [mHAQ], pain, fatigue, patient global assessment, EuroQol 5 dimensions [EQ-5D] index and morning stiffness) from index visit to Month 12 in PSM patients. Subgroup analyses were conducted in patients who were anti-CCP+ at index visit.

## Results

SS data were available for 36,256 patients with RA, of whom 1901 met the inclusion criteria (Fig. 1). There were 454 pairs of patients randomly generated with either RA and SS or RA only by frequency matching based on RA duration (Fig. 1 and Supplementary Table S1). Due to residual cohort imbalance after frequency matching (Supplementary appendix), the frequency-matched cohorts were pooled and further refined by PSM. Of the 454 pairs of frequency-matched patients, 283 pairs of patients were PSM (Table 1 and Fig. 1). The baseline demographics, disease characteristics and PROs of PSM patients with RA, with and without SS, were generally well balanced. At baseline, 62–64% of patients were being treated with tumor necrosis factor inhibitors, 21–22% with other b/tsDMARDs and 16–17% with abatacept. At baseline, 56–59% of patients in each cohort were receiving methotrexate monotherapy.

**Table 1:** Baseline characteristics of the PSM RA cohort

	Patients with RA and SS (*n* = 283)	Patients with RA only (*n* = 283)	*p* value
Age, years, mean (SD)	58.4 (12.2)	58.7 (12.8)	0.712
Sex, female	235 (83.0)	235 (83.0)	1.000
Work status			0.945
Full-time	103 (36.4)	107 (37.8)	
Part-time	33 (11.7)	29 (10.2)	
Disabled	42 (14.8)	46 (16.3)	
Retired	84 (29.7)	83 (29.3)	
Other	21 (7.4)	18 (6.4)	
BMI, kg/m^2^, mean (SD)	30.2 (7.0)	30.5 (7.1)	0.699
Duration of RA, years, mean (SD)	10.5 (10.0)	10.3 (9.7)	0.834
Co-morbidities			
Hypertension	89 (31.4)	91 (32.2)	0.857
CV disease^a^	32 (11.3)	31 (11.0)	0.894
Malignancy^b^	29 (10.2)	31 (11.0)	0.785
Diabetes	27 (9.5)	25 (8.8)	0.771
Serious infections^c^	24 (8.5)	24 (8.5)	1.000
Asthma	18 (6.4)	15 (5.3)	0.590
COPD	5 (1.8)	7 (2.5)	0.560
ILD/pulmonary fibrosis	4 (1.4)	1 (0.4)	0.178
Anti-CCP+, n/m (%)	56/114 (49.1)	66/119 (55.5)	0.333
RF+, n/m (%)	91/137 (66.4)	80/144 (55.6)	0.062
Erosive disease, n/m (%)	74/216 (34.3)	64/197 (32.5)	0.703
Subcutaneous nodules, n/m (%)	69/283 (24.4)	73/283 (25.8)	0.698
Current b/tsDMARDs use	283 (100.0)	283 (100.0)	
TNFi	180 (63.6)	174 (61.5)	0.602
Other b/tsDMARD	59 (20.8)	62 (21.9)	0.758
Abatacept	44 (15.5)	47 (16.6)	0.731
Concomitant csDMARD use, n/m (%)	207/283 (73.1)	213/283 (75.3)	0.848
MTX only	121 (58.5)	119 (55.9)	
Non-MTX csDMARD only	41 (19.8)	40 (18.8)	
MTX and non-MTX combination	39 (18.8)	46 (21.6)	
Non-MTX and csDMARD combination	6 (2.9)	8 (3.8)	
Number of prior b/tsDMARDs			0.794
0	116 (41.0)	121 (42.8)	
1	77 (27.2)	70 (24.7)	
≥ 2	90 (31.8)	92 (32.5)	
Number of prior csDMARDs			0.871
0	16 (5.7)	16 (5.7)	
1	116 (41.0)	110 (38.9)	
≥ 2	151 (53.4)	157 (55.5)	
CDAI score, mean (SD)	24.0 (14.9)	24.1 (14.7)	0.922
PROs			
mHAQ, mean (SD)	0.5 (0.5)	0.6 (0.5)	0.389
Patient pain, mean (SD)^d^	50.6 (27.0)	52.3 (27.3)	0.464
Patient fatigue, mean (SD)^e^	53.6 (29.3)	56.2 (28.9)	0.299
Patient global assessment, mean (SD)	47.8 (26.1)	50.0 (25.4)	0.303
EQ-5D index, mean (SD)^f^	0.7 (0.2)	0.7 (0.2)	0.414
Morning stiffness time, minutes, mean (SD)^g^	111.1 (199.1)	112.3 (195.5)	0.942

Data are *n* (%) unless otherwise stated

^a^History of coronary artery disease, myocardial infarction, congestive heart failure requiring hospitalization, acute coronary syndrome, unstable angina, cardiac revascularization procedure, cardiac arrest, ventricular arrhythmia, stroke, transient ischemic attack, or other CV event

^b^History of lung cancer, breast cancer, lymphoma, skin cancer (melanoma and squamous cell carcinoma), or other cancer

^c^Infection required hospitalization or IV treatment

^d^Patients with RA and SS, *n* = 282

^e^Patients with RA and SS, *n* = 271; patients with RA only, *n* = 237

^f^Patients with RA and SS, *n* = 262; patients with RA only, *n* = 228

^g^Patients with RA and SS, *n* = 251; patients with RA only, *n* = 247

*BMI* body mass index, *b/tsDMARD* biologic or targeted synthetic disease-modifying antirheumatic drug, *CCP+* cyclic citrullinated peptide positive, *CDAI* Clinical Disease Activity Index; *COPD* chronic obstructive pulmonary disease, *csDMARD* conventional synthetic disease-modifying antirheumatic drug, *CV* cardiovascular, *EQ-5D*, EuroQol 5 dimension, *ILD* interstitial lung disease, *IV* intravenous, *mHAQ* modified Health Assessment Questionnaire, *MTX* methotrexate, *n/m* number of patients/total number of patients with available data, *PRO* patient-reported outcome, *RA* rheumatoid arthritis, *RF+* rheumatoid factor positive, *SD* standard deviation, *SS* Sjögren’s syndrome, *TNFi* tumor necrosis factor inhibitor

### RA disease activity

**Fig. 2:**
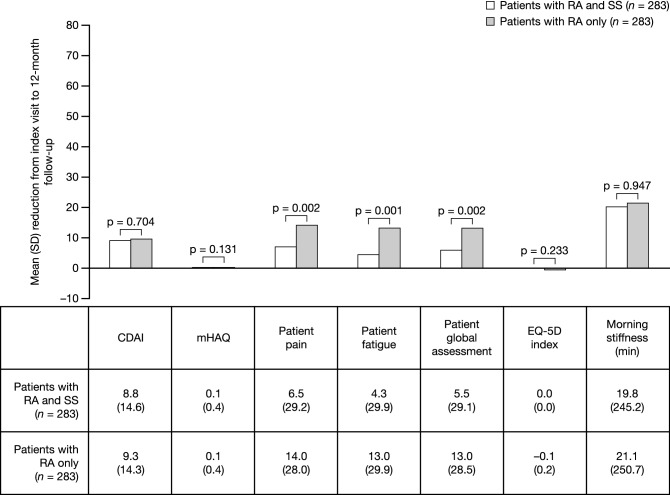
Mean (SD) reduction in CDAI score and PROs from index visit to 12-month follow-up visit. *p* values estimated using Student’s *t* test and Chi-square test. *CDAI* Clinical Disease Activity Index, *EQ-5D* EuroQol 5-dimension, *mHAQ* modified Health Assessment Questionnaire, *PRO* patient-reported outcome, *RA* rheumatoid arthritis, *SD* standard deviation, *SS* Sjögren’s syndrome

At the 12-month follow-up, mean (standard deviation [SD]) CDAI score was numerically higher in patients with RA and SS compared with patients with RA only (Table 2). At the 12-month follow-up, the mean (SD) change in CDAI score from the index visit was numerically lower in patients with RA and SS compared to those with RA only (Fig. 2). These comparisons did not reach statistical significance.

**Table 2:** Mean CDAI score and PROs at the 12-month follow-up

	Patients with RA and SS (*n* = 283)	Patients with RA only (*n* = 283)	*p* value
CDAI	15.2 (13.4)	14.8 (12.8)	0.758
mHAQ	0.5 (0.5)	0.5 (0.5)	0.629
Patient pain	44.2 (29.0)	38.3 (28.1)	0.014
Patient fatigue	49.2 (30.1)	43.4 (28.5)	0.018
Patient global assessment	42.4 (27.8)	37.1 (27.4)	0.023
EQ-5D index	0.7 (0.2)	0.7 (0.2)	0.982
Morning stiffness (min)	92.9 (172.7)	90.6 (209.0)	0.922

Data are mean (SD)

*CDAI* Clinical Disease Activity Index, *EQ-5D* EuroQol 5-dimension, *mHAQ* modified Health Assessment Questionnaire, *PRO* patient-reported outcome, *RA* rheumatoid arthritis, *SD* standard deviation, *SS* Sjögren’s syndrome

### Patient-reported outcomes

At baseline, patients with RA and SS had lower mean (SD) PRO scores than patients with RA only (Table 1). However, at the 12-month follow-up visit, mean (SD) PRO scores were higher in patients with RA and SS compared with patients with RA only (Table 2), with the exception of mHAQ and EQ-5D index scores, which were similar in both cohorts. Mean (SD) patient pain, patient fatigue, and patient global assessment at the 12-month follow-up visit were significantly higher in patients with RA and SS compared with patients with RA only (*p* = 0.014, *p* = 0.018 and *p* = 0.023, respectively). Morning stiffness at 12 months was numerically higher in patients with RA and SS compared with patients with RA only, but this was not statistically significant. Mean changes from the index visit in pain, fatigue and patient global assessment were statistically significant (*p* = 0.002, *p* = 0.001 and *p* = 0.002, respectively), and two- to three-fold inferior (suggesting more impairment), for patients with RA and SS compared with patients with RA only (Fig. 2). The mean change in morning stiffness was numerically lower in patients with RA and SS compared with patients with RA only. The mean change in mHAQ was similar in both cohorts. There was no change in EQ-5D index score from index event until 12-month follow-up for patients with RA and SS, while the mean change for patients with RA only was marginally worse at 12 months.

### *Subgroup analyses in patients with anti-CCP*+ *RA*

**Fig. 3:**
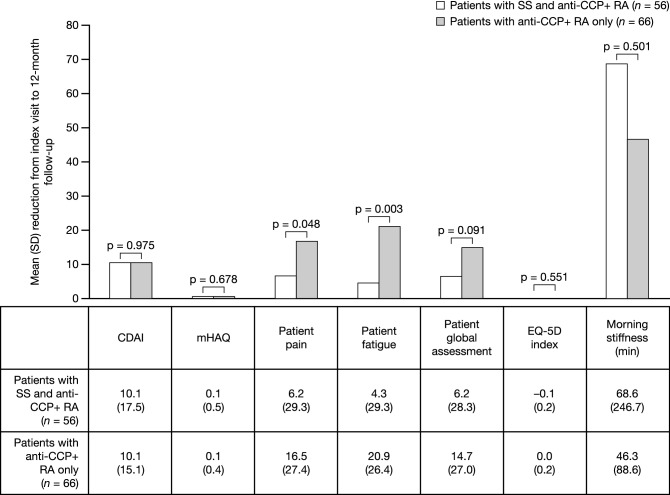
Mean (SD) reduction in CDAI score and PROs from index visit to 12-month follow-up visit in the subgroup of patients with anti-CCP+ RA. *p* values estimated using Student’s *t* test and Chi-square test. *CCP+* cyclic citrullinated peptide positive, *CDAI* Clinical Disease Activity Index, *mHAQ* modified Health Assessment Questionnaire, *PRO* patient-reported outcome, *RA* rheumatoid arthritis, *SD* standard deviation, *SS* Sjögren’s syndrome

Of the 283 PSM pairs, there were 122 patients who were anti-CCP+: 56 (45.9%) patients with RA and SS and 66 (54.1%) patients with RA only (Fig. 1). In patients who were anti-CCP+, the mean (SD) change in CDAI score was similar between patients with RA and SS and those with RA only (Fig. 3). In patients who were anti-CCP+, the mean (SD) change in pain and fatigue was significantly lower in patients with RA and SS compared with patients with RA only (*p* = 0.048 and *p* = 0.003, respectively; Fig. 3). Although numerical differences were observed, there were no statistically significant differences in mean improvements in patient global assessment and morning stiffness outcomes between patients with anti-CCP+ RA and SS and those with anti-CCP+ RA only. In patients who were anti-CCP+, the mean change in mHAQ was the same in both cohorts; there was no change in EQ-5D index score from index event until 12 month follow-up for patients with RA only, while the mean change for patients with RA and SS was marginally worse at 12 months.

## Discussion

In this analysis of data from the large US Corrona RA registry, we compared RA disease activity and PROs among patients with RA, with and without SS, who initiated b/tsDMARD treatment. Initially, patients with RA and SS or RA only were frequency matched by duration of RA. Significantly fewer patients with RA and SS were in full-time employment and more were disabled or retired compared with patients with RA only. Patients with RA and SS were also more likely to have co-morbidities and to be taking several treatments for RA. These results complement published data examining the effect of SS on quality of life [8, 19]. The symptoms of SS exert a burden on a patient that may impact physical and social functioning, which in turn may affect employment status. Combined with the burden of RA [20], SS may substantially affect a patient’s quality of life.

There is a paucity of published data investigating disease activity in patients with RA and SS, particularly from studies measuring cumulative disease activity with index dates defined by SS onset [6, 9, 15, 21–23]; only two of the aforementioned studies included a follow-up period [6, 9]. The studies vary in size (82–1471 patients) [9, 22] and methods used for SS diagnosis, often utilizing a mixture of objective (e.g., Schirmer’s test) and subjective methods (e.g., physician exam and/or questionnaire from patient-reported symptoms) [21–23]. Two cross-sectional observational studies found no relationship between SS status and RA disease activity, as measured by Disease Activity Score 28 (DAS28) using erythrocyte sedimentation rate or C-reactive protein, in patients with RA with and without SS [22, 23]. It should be noted that the proportion of patients with RA and SS in these studies was small: *n* = 20/82 [22] and *n* = 11/307 [23]. In contrast, several studies have noted an association between SS and RA disease activity [9, 15, 21]. The analysis of 1471 patients with RA enrolled in a longitudinal RA registry revealed that patients with RA and SS (*n* = 415) experienced a longer duration of disease, higher RA disease activity (measured by RA disease activity index, CDAI and DAS28) at baseline and significantly lower reduction in RA disease activity at 12 months than patients with RA only [9]. In a study of 636 patients with RA (*n* = 232 patients with SS symptoms), DAS28 was significantly higher for patients with moderate and severe symptoms compared with patients with no symptoms of SS [21]. Similarly, among 509 patients with RA (*n* = 74 patients with RA and SS), patients with RA and SS had significantly higher DAS28 scores than patients with RA only [15].

To minimize selection bias in the current study, patients were PSM to account for variables associated with treatment response. The resulting cohorts (patients with RA and SS and patients with RA only) were generally well balanced in terms of baseline characteristics. At the 12-month follow-up visit, there were no significant differences in the change in clinical disease activity between patients with RA and SS and those with RA only; however, change in CDAI score after 12 months was numerically lower (suggesting greater impairment) in patients with RA and SS compared to those with RA only. The lack of association between SS status and RA disease activity in the present study is likely due to PSM; characteristics that are commonly seen with SS, and that are associated with RA disease activity, were accounted for in this analysis, but may not have been in the aforementioned studies, which did not use PSM [9, 15, 21].

Previous studies have demonstrated that patients with SS only (as opposed to patients with SS co-existing with another autoimmune condition) experience pain and fatigue [24], as do patients with RA only [25]; therefore, it could reasonably be inferred that, when co-existing with RA, SS exacerbates these PROs. In this analysis, at the 12-month follow-up visit, improvements in the majority of RA-related PROs were reduced in patients with RA and SS versus patients with RA only (suggesting more impairment). In accordance with this, several studies have demonstrated the additional burden of SS on patients with RA [9, 21, 23]. In an observational study, patients with RA with moderate or severe SS symptoms had significantly worse scores for mHAQ, pain and fatigue compared with patients with RA with no SS symptoms [21]. In a longitudinal registry analysis, patients with RA and SS experienced significantly higher multidimensional (MD) HAQ fatigue scores at baseline compared with patients with RA only [9]. After 12 months, patients with RA and SS experienced numerically lower reductions in MDHAQ fatigue scores than patients with RA only [9]. In addition, an observational study demonstrated that patients with RA and SS experienced numerically greater pain (visual analog scale) scores compared with patients with RA only [23]. Interestingly in our analysis, the differences in two PRO measures, mHAQ and EQ-5D index, between patients with RA and SS and patients with RA only, were marginal. However, the mHAQ and EQ-5D index questionnaires are completed by patients and would likely be impacted by underlying RA disease status.

Patients with RA who are ACPA+ are more likely to have a more severe disease course than patients who are ACPA− [13]. A study of Greek patients with RA concluded that the presence of anti-CCP antibodies was associated with extra-articular manifestations, such as serositis and pulmonary fibrosis [14]. In several studies, the proportion of patients with anti-CCP antibodies has been found to be higher in patients with RA and SS compared with patients with RA only (67.6–77.8% versus 59.4–71.9%) [6, 9, 15]. Therefore, we sought to clarify if the presence of SS would exacerbate the disease course further in anti-CCP+ patients. Contrary to previous research [6, 9, 15], in the current study, the proportion of patients with anti-CCP antibodies was lower in patients with RA and SS compared with patients with RA only (49.1% versus 55.5%); however, it should be noted that not all patients had measures reported. RA disease activity and PRO results were similar in the subgroup of patients with RA who were anti-CCP+, indicating that SS status did not affect outcomes differently in this patient population.

The biologic pathways associated with the differences in patients with RA with, and without, dry eyes and mouth (SS) are not clear [26–28]. However, the results of the current study do suggest that physicians may wish to consider SS status in the management of patients with RA; in particular, there may be a need for closer monitoring and assessment of response to treatment. More aggressive treatment of SS with RA may be needed.

This analysis has several strengths, namely the Corrona registry is the largest disease registry in the US that collects data directly from both providers and patients at the time of a routine clinical encounter. This allowed for patients with RA and SS or RA only to be selected from a broad population, ensuring satisfactory pairing [29]. The Corrona registry does include a wide variety of rheumatology practices participating throughout the country (rural and urban areas, academic and private settings) and brings access to broad geographic locations and patients with diverse sociodemographic origins. Prior analyses compared Medicare patients with RA enrolled in Corrona to those who are not part of the registry and found similar demographic and co-morbidity characteristics, supporting the generalizability of the Corrona registry [29]. Data are collected at regular intervals, which enabled us to evaluate outcomes at two different time points. Advanced epidemiological methods (e.g., PSM) were used to compare responses between patients with and without SS where selection bias may have existed. The presence of SS was captured on the provider form and is considered a critical field of data for Corrona. The results from this observational study in US patients complement previous studies [8, 9, 19, 21–23].

As opposed to previously reported studies, bias was minimized by PSM of the two cohorts (RA and SS versus RA only) by factors known to be associated with treatment response (e.g., age, sex, CDAI score, number of prior biologics, work status, history of co-morbidities and RA disease characteristics). Another limiting factor was that patients included in the registry were diagnosed by different rheumatologists across the US; diagnosis of SS was at the discretion of the treating physician (SS associated with RA: yes/no) and rheumatologists may have used a variety of mostly historical clinical signs and symptoms rather than objective testing (i.e., Schirmer’s test). Nevertheless, we believe that the manner in which these data were collected reflects real-world clinical practice with the reporting of dry eyes and/or mouth not associated with medications or mouth breathing during the night. Additionally, patients in the RA-only cohort also required a questionnaire entry for SS associated with RA (yes/no), potentially leading to under-ascertainment.

In this large US patient population initiating b/tsDMARD treatment, patients with RA only had greater improvements in RA disease activity and PROs than those with RA and SS; similar results were shown in anti-CCP+ patients. Physicians may wish to consider SS status when managing patients with RA; specifically, patients with RA and SS may require closer monitoring and more aggressive intervention to improve their disease experience than patients with RA only. Additional studies are needed to further understand the biological pathways involved in SS co-existing with RA, and subsequently to provide targeted treatment options for this population of patients.

## Electronic supplementary material

Below is the link to the electronic supplementary material.

Supplementary file1 (DOCX 27 kb)

## Data Availability

The datasets generated and/or analyzed during the current study are not publicly available, but are available from the corresponding author upon reasonable request. The Corrona dataset is based on a large US multicenter study adhering to a number of institutional review boards, with complex logistics. Patients did not provide consent to raw data sharing during the data collection for this purpose, and the Corrona data-sharing policies do not permit raw data sharing for this purpose. An aggregated limited dataset from the current analyses is available to qualified investigators with an approved protocol. Data requests may be sent to Corrona, represented by Dr. Jeffrey D. Greenberg MD MPH, NYU School of Medicine, New York, NY, e-mail jgreenberg@corrona.org.
