# Supplementary material for: Disease activity and patient-reported outcomes in patients with rheumatoid arthritis and Sjögren’s syndrome enrolled in a large observational US registry
Source: Rheumatol Int. 2020 May 24;40(8):1239–48. doi: 10.1007/s00296-020-04602-8 (PMC7316680; doi:10.1007/s00296-020-04602-8)
Supplement: Supplementary file 1 — Supplementary file1 (DOCX 27 kb) [file 296_2020_4602_MOESM1_ESM.docx]

## SUPPLEMENTARY APPENDIX

This appendix has been provided by the authors to give readers additional information about their work.

# Supplement to: Rheumatoid arthritis disease activity and patient-reported outcomes in patients with rheumatoid arthritis and Sjögren’s syndrome enrolled in a large observational US registry (*Rheumatology International*)

Leslie R. Harrold • Ying Shan • Sabrina Rebello • Neil Kramer • Sean E. Connolly • Evo Alemao •

Sheila Kelly • Joel M. Kremer • Elliot D. Rosenstein

**Corresponding author:**

Leslie R. Harrold

Corrona, LLC, Waltham, MA, USA and University of Massachusetts Medical School, Worcester, MA, USA

E-mail: [lharrold@corrona.org](mailto:lharrold@corrona.org)

**Contents:**

**Results**

*Frequency matching*

**Supplementary tables**

*Table S1. Baseline characteristics of the frequency-matched RA cohort*

**Results**

*Frequency matching*

Patients with rheumatoid arthritis (RA) and Sjögren’s syndrome (SS) were statistically significantly more likely to have an impaired work status and to have hypertension (p = 0.003), cardiovascular disease (p = 0.005) or diabetes (p <0.001) compared with patients with RA only (Table S1). Patients with RA and SS were statistically significantly less likely to be treated with a tumour necrosis factor inhibitor (TNFi) biologic (p = 0.008) and more likely to be treated with a non-TNFi biologic or targeted synthetic disease-modifying antirheumatic drug (b/tsDMARD; p = 0.001; ≥2 b/tsDMARDs; p = 0.002) or ≥2 conventional synthetic DMARDs (p = 0.003) compared with patients with RA only (Table S1).

**Table S1** Baseline characteristics of the frequency-matched RA cohort

|  | Patients with RA and SS  (*n* = 454) | Patients with RA only  (*n* = 454) | p value |
| --- | --- | --- | --- |
| Age, years, mean (SD) | 60.1 (12.2) | 57.4 (12.5) | 0.001 |
| Sex, female | 392 (86.3) | 346 (76.2) | <0.001 |
| Work status^a^ |  |  | 0.003 |
| Full-time | 132 (29.7) | 182 (40.7) | 0.001 |
| Part-time | 43 (9.7) | 46 (10.3) | 0.754 |
| Disabled | 92 (20.7) | 66 (14.8) | 0.021 |
| Retired | 149 (33.5) | 119 (26.6) | 0.025 |
| Other | 29 (6.5) | 34 (7.6) | 0.525 |
| BMI, kg/m^2^, mean (SD) | 30.5 (7.5) | 30.3 (7.2) | 0.711 |
| Duration of RA, years, mean (SD) | 10.8 (10.1) | 10.7 (9.9) | 0.809 |
| Co-morbidities |  |  |  |
| Hypertension | 165 (36.3) | 123 (27.1) | 0.003 |
| CV disease^b^ | 66 (14.5) | 39 (8.6) | 0.005 |
| Malignancy^c^ | 45 (9.9) | 41 (9.0) | 0.650 |
| Diabetes | 64 (14.1) | 31 (6.8) | <0.001 |
| Serious infections^d^ | 43 (9.5) | 30 (6.6) | 0.113 |
| Asthma | 26 (5.7) | 19 (4.2) | 0.284 |
| COPD | 8 (1.8) | 16 (3.5) | 0.098 |
| ILD/pulmonary fibrosis | 5 (1.1) | 2 (0.4) | 0.255 |
| Anti CCP+, n/m (%) | 97/187 (51.9) | 103/182 (56.6) | 0.363 |
| RF+, n/m (%) | 157/232 (67.7) | 134/221 (60.6) | 0.118 |
| Erosive disease, n/m (%) | 130/358 (36.3) | 107/309 (34.6) | 0.650 |
| Subcutaneous nodules, n/m (%) | 153/454 (33.7) | 97/454 (21.4) | 0.000 |
| Current b/tsDMARDs use | 454 (100.0) | 454 (100.0) |  |
| TNFi | 250 (55.1) | 289 (63.7) | 0.008 |
| Other b/tsDMARD | 127 (28.0) | 84 (18.5) | 0.001 |
| Abatacept | 77 (17.0) | 81 (17.8) | 0.726 |
| Concomitant csDMARD use, n/m (%) | 331/454 (72.9) | 338/454 (74.4) | 0.533 |
| MTX only | 191 (57.7) | 200 (59.2) |  |
| Non-MTX csDMARD only | 69 (20.8) | 66 (19.5) |  |
| MTX and non-MTX combination | 50 (15.1) | 58 (17.2) |  |
| Non-MTX and csDMARD combination | 21 (6.3) | 14 (4.1) |  |
| Number of prior b/tsDMARDs |  |  | 0.003 |
| 0 | 164 (36.1) | 207 (45.6) | 0.004 |
| 1 | 116 (25.6) | 117 (25.8) | 0.939 |
| ≥2 | 174 (38.3) | 130 (28.6) | 0.002 |
| Number of prior csDMARDs |  |  | 0.010 |
| 0 | 26 (5.7) | 36 (7.9) | 0.188 |
| 1 | 161 (35.5) | 196 (43.2) | 0.017 |
| ≥2 | 267 (58.8) | 222 (48.9) | 0.003 |
| CDAI score, mean (SD) | 24.2 (15.2) | 24.1 (14.2) | 0.868 |
| PROs | | | |
| mHAQ, mean (SD)^e^ | 0.6 (0.5) | 0.5 (0.5) | 0.014 |
| Patient pain, mean (SD)^f^ | 52.5 (27.6) | 51 (27.8) | 0.429 |
| Patient fatigue, mean (SD)^g^ | 54.7 (29.6) | 53.7 (30.1) | 0.632 |
| Patient global assessment, mean (SD)^h^ | 49.6 (26.9) | 48 (26.2) | 0.379 |
| EQ-5D index, mean (SD)^i^ | 0.7 (0.2) | 0.7 (0.2) | 0.281 |
| Morning stiffness time, minutes, mean (SD)^j^ | 120 (204.8) | 107.5 (185.3) | 0.341 |

Data are n (%) unless otherwise stated

^a^Patients with RA and SS, *n* = 445; patients with RA only, *n* = 447

^b^History of coronary artery disease, myocardial infarction, congestive heart failure requiring hospitalization, acute coronary syndrome, unstable angina, cardiac revascularization procedure, cardiac arrest, ventricular arrhythmia, stroke, transient ischemic attack or other CV event

^c^History of lung cancer, breast cancer, lymphoma, skin cancer (melanoma and squamous cell carcinoma) or other cancer

^d^Infection required hospitalization or IV treatment

^e^Patients with RA and SS, *n* = 441; patients with RA only, *n* = 449

^f^Patients with RA and SS, *n* = 452; patients with RA only, *n* = 451

^g^Patients with RA and SS, *n* = 428; patients with RA only, *n* = 387

^h^Patients with RA and SS, *n* = 453; patients with RA only, *n* = 451

^i^Patients with RA and SS, *n* = 406; patients with RA only, *n* = 371

^j^Patients with RA and SS, *n* = 390; patients with RA only, *n* = 388

*BMI* body mass index, *b/tsDMARD* biologic or targeted synthetic disease-modifying antirheumatic drug, *CCP+* cyclic citrullinated peptide positive, *CDAI* Clinical Disease Activity Index, *COPD* chronic obstructive pulmonary disease, *csDMARD* conventional synthetic disease-modifying antirheumatic drug, *CV* cardiovascular, *EQ-5D* EuroQol 5-dimension, *ILD* interstitial lung disease, *IV* intravenous, *mHAQ* modified Health Assessment Questionnaire; *MTX* methotrexate, n/m, number of patients/total number of patients with available data; PRO, patient-reported outcomes; *RA* rheumatoid arthritis, *RF+* rheumatoid factor positive, *SD* standard deviation, *SS* Sjögren’s syndrome, *TNFi* tumour necrosis factor inhibitor
